# 8-(Pyridin-2-yl)quinolin-7-ol as a Platform for Conjugated Proton Cranes: A DFT Structural Design

**DOI:** 10.3390/mi11100901

**Published:** 2020-09-29

**Authors:** Anton Georgiev, Liudmil Antonov

**Affiliations:** 1 Department of Organic Chemistry, University of Chemical Technology and Metallurgy, 1756 Sofia, Bulgaria; antonchem@abv.bg; 2 Institute of Optical Materials and Technologies, Bulgarian Academy of Sciences, 1113 Sofia, Bulgaria; 3 Institute of Electronics, Bulgarian Academy of Sciences, 1784 Sofia, Bulgaria

**Keywords:** proton crane, 7-hydroxyquinoline, DFT, proton transfer, tautomerism, ESIPT

## Abstract

Theoretical design of conjugated proton cranes, based on 7-hydroxyquinoline as a tautomeric sub-unit, has been attempted by using ground and excited state density functional theory (DFT) calculations in various environments. The proton crane action request existence of a single enol tautomer in ground state, which under excitation goes to the excited keto tautomer through a series of consecutive excited-state intramolecular proton transfer (ESIPT) steps with the participation of the crane sub-unit. A series of substituted pyridines was used as crane sub-units and the corresponding donor-acceptor interactions were evaluated. The results suggest that the introduction of strong electron donor substituents in the pyridine ring creates optimal conditions for 8-(pyridin-2-yl)quinolin-7-ols to act as proton cranes.

## 1. Introduction

Excited state proton transfer reactions involve proton transfer through a ground state pre-existing hydrogen bond (either intra- or intermolecular), giving rise to another tautomer in the excited state [1,2,3,4,5]. Due to dramatic structural change, the excited tautomer possesses photophysical properties, different from those of the ground state specie. The ESIPT exhibiting molecules have become a field of active research [6] in the last decades, due to their applications as light-emitting materials and laser dyes [7,8,9], photo stabilizers [10] and photo switches [11,12].

**Scheme 1:**
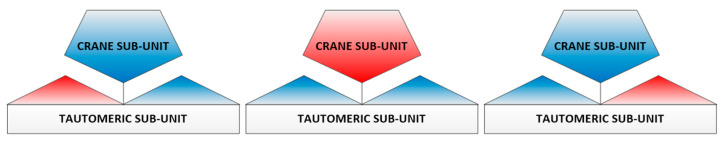
Proton crane in *standby*, *transfer* and *end* states (from left to right). The proton donor parts are given in red, while the proton acceptors are marked in blue.

Proton cranes are switching systems where a proton is transferred over a long distance within the same molecule. In general they contain a tautomeric sub-unit with clearly defined proton donor and proton acceptor parts, which exchange, under external stimuli, the tautomeric (or pseudo tautomeric) proton using a crane sub-unit as a proton delivery system. The overall process is sketched in Scheme 1, showing the sub-units linked by an axle. The proton is located in the tautomeric sub-unit in the single, solely existing, stable *standby* state. Under suitable irradiation, proton transfer occurs from the tautomeric to the crane sub-unit, leading to the unstable *transfer* state. Depending on the relative proton acceptor ability of the two competitive sites in the deprotonated tautomeric sub-unit, the protonated crane sub-unit might or might not rotate around the axle, leading, after release of the proton, to the *end* or to the *standby* state, correspondingly. The obtained *end* state can relax back to the *standby* either thermally or through applying another stimulus (another excitation wavelength for instance). The process of switching from the *standby* to the *end* state and back can be monitored easily by optical spectroscopy due to the different absorption spectra of the tautomers [2]. Although the rotation around the axle is not unidirectional, the proton cranes can be considered as simple prototypes of molecular motors [13] and, therefore deserve detailed investigation. Some of them, being named “small-molecule robotic arms”, have been used to deliver “cargo” from one side of the molecule to another [14].

**Scheme 2:**
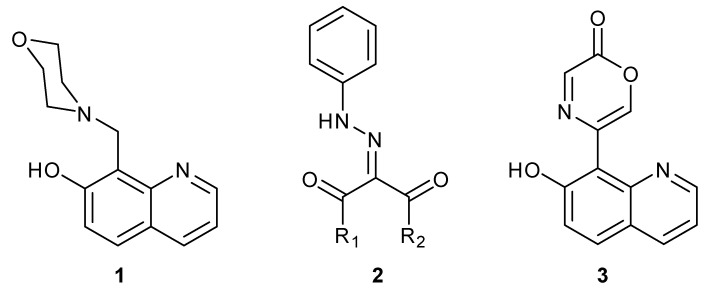
Examples for proton cranes: single-bond axle non-conjugated (**1**) and conjugated (**3**), double-bond axle (**2**).

Based on the structural design, the proton cranes can be classified depending on: (a) single- or double-bond character of the axle; (b) extent of conjugation between the tautomeric and crane sub-units; (c) heteroatoms being used as proton donor and proton acceptor sites. Several classes of compound could be mentioned as typical examples. Historically, the term “proton crane” was used for the first time by Varma et al. [15,16,17,18], who designed a single bond axle system, 8-(morpholinomethyl)quinolin-7-ol (**1** in Scheme 2), where the proton is exchanged between O and N in the 7-hydroxyquinoline upon irradiation [19]. Proton crane **1** and its mimics [12,20,21] are based on an excited state proton transfer pathway upon irradiation as the only possibility to reach the energetically unfavorable *end* state. Another intriguing concept is the design of systems, where a moderate energy gap between tautomers of the tautomeric sub-unit provides proton exchange in a ground state [12,22,23,24].

In the rotary switches, where the axle is a C=N bond, the proton is transferred between oxygen atoms belonging either to β-dicarbonyl groups (**2** in Scheme 2 as an example) [25,26,27,28] or 4-hydroxycoumarin [29,30,31], or proton transfer proceeds between oxygen and nitrogen in the switches designed by Aprahamian et al. [32,33,34,35]. The rotary switches operate mainly in ground state through acid/base catalyzed tautomerism [32].

**Scheme 3:**
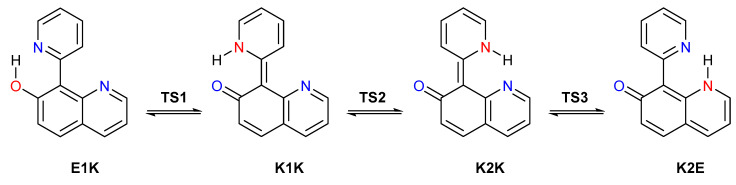
Possible tautomers in **5** given as an example of the studied molecules.

The strongly conjugated proton cranes were theoretically introduced by Sobolewski [36] in the case of 5-(7-hydroxyquinolin-8-yl)-2H-1,4-oxazin-2-one (**3** in Scheme 2). The theoretical design was later expanded by using a variety of tautomeric (7-hydorxyquinolines [37,38,39,40,41,42], 3-OH and 3-NH_2_ pyridines [43]) and crane (carbaldehydes [37,39], carboxamides [40,42], pyridines [38,43] and pyrimidines [41]) sub-units. The experimentally studied systems are limited to a few examples of 7-hydroxyquinoline (**4**) [39], 3-hydroxypyridine [44,45,46,47] and amide [48] tautomeric sub-units. The common feature of these systems is the excited state proton transfer either from E1K* or from K2E* (Scheme 3) to K1K* or K2K* respectively, which results to a conical intersection in the transition state between K1K* and K2K* (tautomeric and crane sub-units being perpendicular). This populates the ground state **K1K** and **K2K**, leading then to restoration of the existing ground state equilibrium over time.

**Scheme 4:**
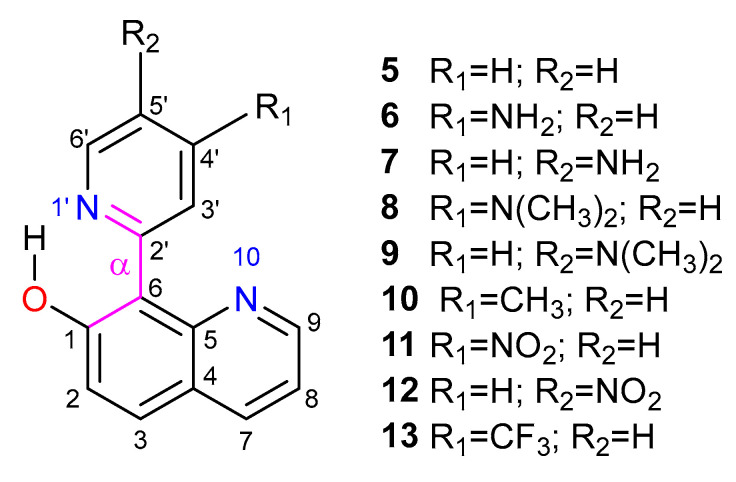
Studied compounds presented as the most stable ground state form. The C_1_C_6_C_2′_N_1′_ dihedral angle is marked as α.

The theoretical design of such proton transfer-based systems faces many challenges related to correct prediction of the ground state tautomerism, solvent effects and excited state dynamics upon irradiation. Recently, we have been able to accumulate knowledge about using density functional theory (DFT) calculations for correct prediction of the ground and excited state tautomerism of dyes and heterocycles [49,50,51,52,53], which gives a good opportunity to attempt the design of proton cranes. 8-(Pyridin-2-yl)quinolin-7-ol, where 7-hydroxy quinoline (**4**) serves as a tautomeric sub-unit, has been selected as a platform to investigate the effect of the substituents in the pyridine crane sub-unit (Scheme 4). The use of **4** as a tautomeric sub-unit gives the advantage of monitoring the switching easily since the *end* keto tautomer absorption is red shifted in respect of the enol *standby* state.

The aim of the study is to investigate the effect of the structural modifications and the solvent of the ground and excited state energy landscape by using DFT calculations and then to define structural conditions needed for a potentially working conjugated proton crane. Although the exited state energy landscape of **5** and **7** has been studied theoretically in the gas phase before [38], the current investigation gives new in-depth information about the possible performance of both ground and excited states and considers the solvent and substituents effects together.

## 2. Theoretical Simulations

Quantum-chemical calculations were performed using the Gaussian 09 D.01 program suite [54]. If not specified otherwise, the M06-2X [55,56] functional with the TZVP [57] basis set were used for the calculations in the ground and excited states, respectively. The M06-2X functional was selected for three reasons. First, it has shown that there is good empirical relations between the relative stability of the tautomers, defined as ΔE, and the experimentally determined ΔG^o^ values of tautomeric azo and azomethyne dyes in cyclohexane and acetonitrile [50,58,59], and of heterocyclic tautomers [49,53]. Second, the use of M06-2X provides very good predictability of the *E/Z* isomerization ratio in some rotary switches [51,60]. Third, the use of this function has given acceptable results in describing the solvent assisted proton transfer in **4** [61,62].

The implicit solvation was described using the polarizable continuum model (the integral equation formalism variant, IEFPCM, as implemented in Gaussian 09) [63]. All ground state structures were optimized without restrictions, using tight optimization criteria and an ultrafine grid in the computation of two-electron integrals and their derivatives. The true minima were verified by performing frequency calculations in the corresponding environment. The transition states were estimated using the Berny algorithm [64] and again verified by performing frequency calculations in the corresponding environment. 

The time dependent DFT (TD-DFT) method [65,66,67] was used for singlet excited state optimizations, again without restrictions, applying tight optimization criteria and an ultrafine grid in the computation of two-electron integrals and their derivatives. The excited state calculations are limited to S_1_, because, as shown in Table S1, the S_2_ state is forbidden, and the proton crane action is linked to the S_1_ landscape according to previous results [38]. In the case of **5** the excited energy landscape was confirmed by using CAM-B3LYP [68]. The absorption spectra of the tautomers were simulated by using B3LYP [69] density functional taking into account its good predictability [67].

The natural bond orbitals (NBO) [70] analysis was performed as implemented in Gaussian 09) [63].

## 3. Results and Discussion

The structure of **4** suggests possibility for enol (**E**) and the keto (**K**) tautomer exchange. However, the large distance between the proton donor (O) and proton acceptor (N) sites makes the direct intramolecular proton transfer (PT) not possible, and thus, solvent-assisted PT is the only possible mechanism for conversion between the tautomers in this molecule [71,72]. Absorption and emission are observed from the **E** tautomer only in non-protic solvents at room temperature [73], whereas in aqueous solution, fluorescence was observed from the **K** form only [74]. Dual emission was observed in alcohols [75,76,77]. In the ground state, the **E** tautomer is the only existing form in non-protic and dry protic solvents, while both tautomers are stable in comparable amounts in the presence of water. Solvent-assisted excited-state proton transfer was proposed, based on the experimental data, in protic solvents [72,76,78], where the process is facilitated by the increased excited state acidity/basicity of the enol/imino groups [79]. It should be noted that concentration effect also plays a substantial role [73,80] in the PT through intermolecular interactions of species. There is a large number of papers where the solvent assisted PT mechanism has been proven by a variety of experimental measurements [81,82,83,84] and theoretical simulations [61,62,85,86].

**Figure 1:**
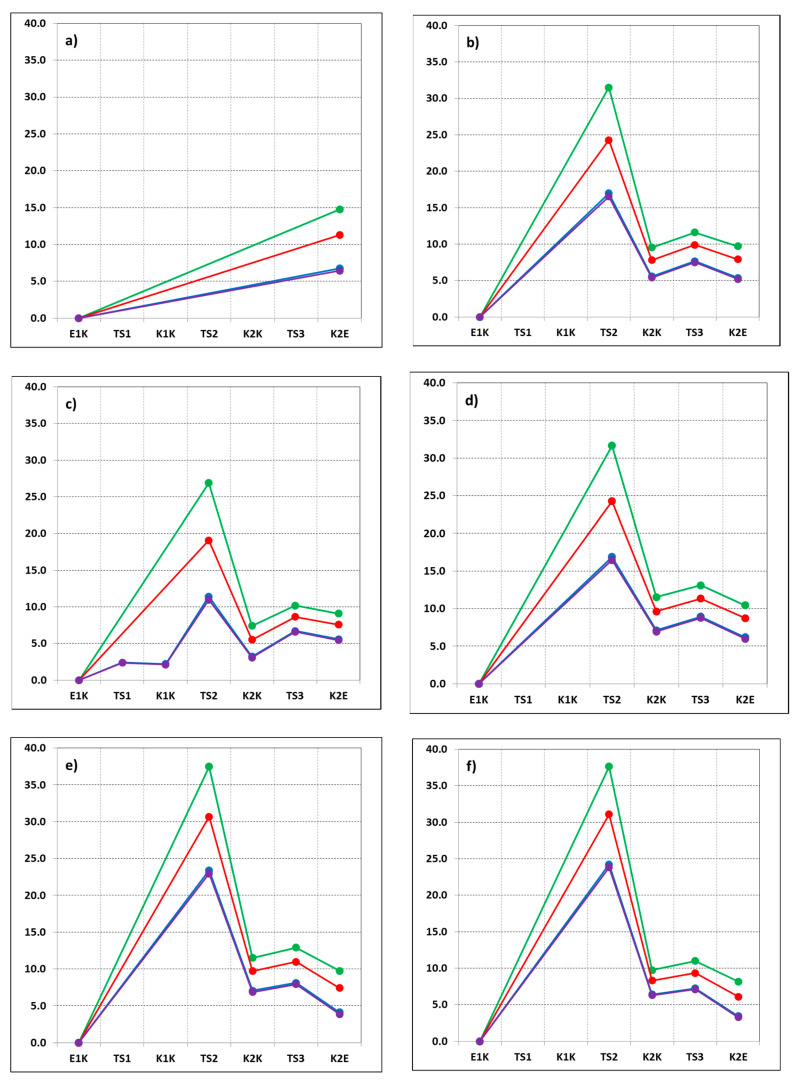
Ground state energy landscape (relative energies in kcal/mol units) of **4**–**7** (**a**–**d**)) and **11**–**12** (**e**,**f**) in vacuum (green), toluene (red), acetonitrile (blue) and formamide (violet). The filled circles represent optimized structures.

**Figure 2:**
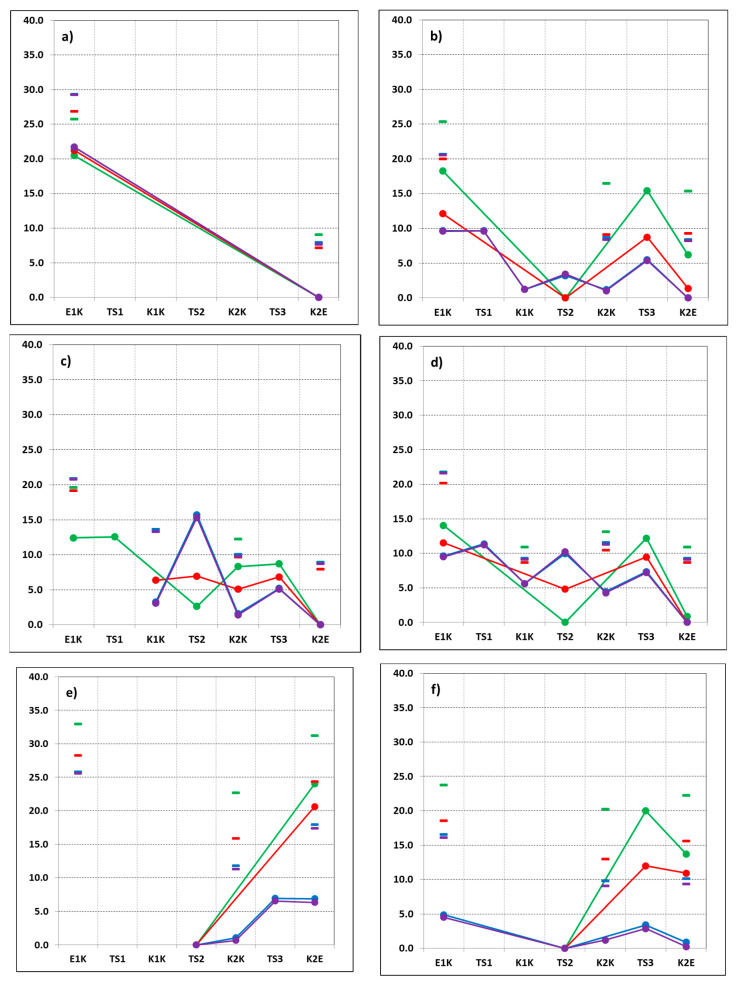
Excited (S_1_) state energy landscape (relative energies in kcal/mol units) of **4**–**7** (**a**–**d**)) and **11**–**12** (**e**,**f**) in vacuum (green), toluene (red), acetonitrile (blue) and formamide (violet). The filled circles represent optimized structures. The corresponding vertical transitions are given in dashes.

The energy landscape of **4** is quite simple and represents an idealistic case for potential PT-based switching. As seen in Figure 1a and Figure 2a, Table 1, and according to the experimental results discussed above, the compound is presented as a single **E** tautomer (**E1K**) in the ground state, which upon excitation (E*) goes through ESPT to the more stable K*, relaxing then to the unstable **K** (**K2E**) and finally reaching again the **E** state through a thermal relaxation. NBO analysis of the tautomeric states, shows donor-acceptor interaction between OH group and quinoline nitrogen with a charge transfer from the enol to the quinoline nitrogen (Table S3, Scheme S1). Sufficient charge transfer in the opposite direction is observed in the keto tautomer. The substantially larger dipole moment of the keto tautomer (Table 2) leads to increased stability in ground state with increasing polarity of the solvent (Figure 1a), but not in an extent to be eventually observed in solution. A similar, but weaker, effect is observed in the excited state. The simulated absorption spectra (Figure S2a) clearly show the advantage of the PT-based switching. The enol tautomer absorbing around 300 nm under suitable condition can give rise of a strongly red shifted keto form.

Another option to make the proton transfer in **4** intramolecular is to attach a crane sub-unit which performs series of consecutive intramolecular PT steps through intramolecular hydrogen bonding (for example, compounds **1** and **3** in Scheme 2) [18,19,36]. In the case of the conjugated proton cranes the overall mechanism includes four tautomers and the corresponding transition states (**TS1**-**3**) between them as sketched in Scheme 3. Correspondingly, the energy landscape in ground and excited state is governed by the energy and relative stability of these structures. The existence of intramolecular hydrogen bonding suggests limited specific solute-solvent interactions (except in **TS2**) and the solvent can be considered as a continuum solvation, affecting the relative stability through dipole moments of the corresponding structures. Therefore, solvents, covering a wide range of dielectric constants, were selected in the current study. Toluene (ε = 2.37) and acetonitrile (ε = 35.6) are aprotic solvents, while formamide (ε = 108.9) is a strongly polar solvent with dualistic proton acceptor and proton donor nature [87,88], selected to provide the upper border of the experimentally affordable dielectric constant. It should be noted that in all studied compounds the major, environmentally defined changes in the energy landscape are observed from a vacuum to toluene and then to acetonitrile. The differences between acetonitrile and formamide are negligible both as shapes and values.

The attachment of the unsubstituted pyridine crane sub-unit (compound **5**) gives interesting information for comparison. Compared with 7-hydroxyquinoline **4**, the atomic charge of the quinoline nitrogen in **5E1K** increases. (Tables S3 and S4) following the increased resonance π-delocalization and charge transfer between the aromatic sub-units. Moreover, the structure is almost planar (Table 2), supporting the effective π-delocalization. Compared to the unsubstituted pyridine, the atomic charge of the nitrogen atom of the crane sub-unit rises in **5**, indicating increased basicity. However, the basicity of the pyridine nitrogen atom remains insufficient to attract the tautomeric proton from the oxygen (no **K1K** form). Further NBO analysis of **5E1K** displays almost negligible donor–acceptor interaction by the quinoline and pyridine sub-units (Scheme S1). According to Table 1 and, as shown in Figure 1b, the enol **E1K** form remains the most stable (single) tautomer. Although some additional stabilization of the *end* state **K2E** is achieved depending on the environment, the values of the relative energies do not suppose its existence in solution before the excitation. Since the **K1K** form is not presented, the ground state PT occurs directly to **K2K**. As seen, the respectively large barrier (perpendicular **TS2**, Figure 1b), due to its large dipole moment (~8D in vacuum), is substantially affected by the solvent environment. The competitive basicity of the nitrogen atoms (Table S2) makes the **K2K** and **K2E** forms almost equally stable with the proton being practically delocalized (negligible **TS3**). The comparable stability of **K2K** and **K2E** does not allow the pure *end* state of switching, as defined in Scheme 1, to be achieved. This conclusion is also confirmed by the NBO analysis of the aforementioned tautomeric states (Scheme S1), showing that the protonated pyridine sub-crane of **K2K** acts as very strong donor in respect of the deprotonated tautomeric sub-unit. Most probably, the charge transfer character of **K2K** is responsible for its stabilization in respect of **K2E**. As mentioned above, the ground state-relative energies of the tautomeric forms indicate that **E1K** is the solely presented form, which can be excited. The excited state energy landscape (Figure 2b) approximates the shape suggested by Sobolewski et al. [36,38] in structurally similar conjugated proton cranes. As an additional verification, the excited energy landscapes obtained by the currently used M06-2X density functional and the CAM-B3LYP one, showing essentially the same changes, are compared in Figure S1. In a gas phase a very fast ESIPT proton transfer from the excited enol leads to a perpendicular minimum and populates both **E1K** and **K2K** in the ground state through a conical intersection. The landscape in toluene is essentially the same. In polar solvents, the ESIPT from E1K* should yield K1K* and eventually K2K* through a very low barrier. The strong red shift of the absorption of **K2K** and **K2E**, comparing to **E1K**, is the overall effect expected in the optical spectra (Figure S2b).

However, the relative stability gradient from K1K* to K2E* does not seem sufficient to achieve the needed ground state population of the latter. This fact and the equal stability of **K2K** and **K2E** make compound **5**, at least according to these simulations, unsuitable for a proton crane. One of the options for improvement is to tune the basicity of the pyridine nitrogen atom through substitution in positions 4′ and 5′ in order to avoid steric hindrance with the tautomeric sub-unit.

The substitution of pyridine crane sub-unit at 4′- and 5′- positions by different electron withdrawing (EW) and electron donating (ED) groups influences the basicity of pyridine nitrogen and is insignificant for the tautomeric sub-unit (Scheme 4) [89]. The 4′-substituent is considered as *para* in respect to the pyridine nitrogen and affects the π-π delocalization depending on its donor-acceptor properties. Compared to the unsubstituted **5**, the donor character of NH_2_ in **6** (+M and +I effects) leads to strong involvement of the nonbonding electron pairs of amine nitrogen to the aromatic π-delocalization. This increases the π-electron density of the ring and the basicity of pyridine nitrogen, which favors the proton attraction form the tautomeric sub-unit. As seen from Figure 1c and Figure 2c, the introduction of an amino (or dimethylamino, Figure S3a) in *para* position in respect of the pyridine nitrogen changes the energy landscape substantially. The increased basicity of the N_1′_ leads to stabilization of the **K1K** form in polar solvents to the extent that it could be observed in acetonitrile/formamide (<1% according to Table 1). The red shift of the **K1K** absorption in respect of **E1K** (Figure S2c) provide conditions for selective excitation even if both are presented simultaneously in solution. The increased basicity effect leads to stabilization of **K2K** in respect of **K2E**. Upon excitation of **E1K** in solution the vertically excited state relaxes to K1K* and then should follow the decreasing energy trend to K2E*. The situation is more favourable in toluene, where the TS2* and TS3* are fairly low, promising a fast switching process. According to Figure S2c the process can be monitored by optical spectroscopy due to the good band separation.

The *meta* (5′) positioned amino group reduces the pyridine nitrogen basicity and changes the relative stability of **K2K** (Figure 1d, Figure S3b for **9**) in comparison with **5**, making the **K2E** form slightly more stable. The excited **E1K** relaxes in solution either directly (in toluene) or through a series of consecutive PT steps (in acetonitrile and formamide) to K2E*. The replacement of the amino with dimethylamino group in position 4′ or 5′ (**8** and **9**) does not change the ground state landscape in comparison with **6** and **7**. The weak σ-donating ED CH_3_ group in **10** by its +I effect leads (Figure S3c) to the same ground state relative stability of the tautomeric forms as in **5**.

As expected, the introduction of electron acceptor group in the crane decreases basicity of the pyridine nitrogen atom (due to the –M effect in **11**–**12**, Scheme S1; and –I effect in **13**) and increases the conjugation between the sub-units, leading to a larger barrier of rotation around the axle. As a result the **K2K** forms in **11**–**13** become less stable in comparison with the **K2E** ones (Figure 1e,f and Figure S3d). As shown in Figure 2e,f, the excitation of the enol tautomer in **11** and **12** leads to a perpendicular TS2* state, which should populate upon relaxation both ground state **E1K** and **K2K**, resulting in low efficiency of switching to **K2E**. In both compounds K2E* forms are substantially less stable in respect of TS2*, reducing the possibility for direct relaxation to the ground *end* state. 

## 4. Conclusions

The ground and excited state energy landscape of conjugated proton cranes based on substituted 8-(pyridin-2-yl)quinolin-7-ols was investigated by means of DFT and TD-DFT calculations in various environments. The results indicate that the appropriate condition for switching requests different tautomeric states of the tautomeric sub-unit to be achieved in ground and in excited states. According to the simulations, such conditions were suggested when the electron donating amine substituent is introduced in the pyridine crane sub-unit. The effect of the solvent also plays a substantial role through stabilization based on the relative polarity of the tautomers.

## Figures and Tables

**Table 1:** Relative energies of the tautomers of **4**–**7** and **11**–**12** in ground and excited S_1_ (in brackets together with the corresponding oscillator strength) state.

Compound	Environment	ΔE, kcal/mol			
		E1K	K1K	K2K	K2E
**4**	vacuum	0.0 (20/0.08)	-	-	15 (0.0/0.08)
	toluene	0.0 (21/0.13)	-	-	11 (0.0/0.15)
	acetonitrile	0.0 (22/0.22)	-	-	6.7 (0.0/0.27)
**5**	vacuum	0.0 (18/0.26)	- (-)	9.6 (-)	9.7 (6.2/0.05)
	toluene	0.0 (12/0.38)	- (-)	7.8 (-)	7.9 (1.37/0.10)
	acetonitrile	0.0 (9.7/0.54)	- (1.22/0.32)	5.6 (1.18/0.52)	5.4 (0.0/0.20)
**6**	vacuum	0.0 (12/0.23)	- (-)	7.4 (8.3/0.23)	9.1 (0.0/0.05)
	toluene	0.0 (-)	- (6.4/0.30)	5.5 (5.1/0.34)	7.6 (0.0/0.10)
	acetonitrile	0.0 (-)	2.2 (3.3/0.52)	3.2 (1.6/0.44)	5.6 (0.0/0.19)
**7**	vacuum	0.0 (14/0.30)	- (-)	11 (-)	10 (0.86/0.04)
	toluene	0.0 (11/0.46)	- (-)	9.6 (-)	8.7 (0.0/0.07)
	acetonitrile	0.0 (9.6/0.68)	- (5.6/0.37)	7.1 (4.4/0.55)	6.2 (0.0/0.15)
**11**	vacuum	0.0 (-)	- (-)	11 (-)	9.7 (24/0.14)
	toluene	0.0 (-)	- (-)	9.7 (-)	7.4 (21/0.13)
	acetonitrile	0.0 (-)	- (-)	7.0 (1.06/0.24)	4.1 (6.8/0.15)
**12**	vacuum	0.0 (-)	- (-)	9.7 (-)	8.1 (14/0.06)
	toluene	0.0 (-)	- (-)	8.3 (-)	6.1 (11/0.17)
	acetonitrile	0.0 (4.9/0.88)	- (-)	6.4 (1.20/0.87)	3.4 (0.88/0.85)

**Table 2:** Important structural parameters of the tautomers of **4**–**7** and **11**–**12** in ground and excited S_1_ (in brackets) state.

Compound	Environment	E1K	K1K	K2K	K2E
Dipole moment, in D					
**4**	vacuum/ acetonitrile	1.2/1.8 (1.3/1.9)			7.9/12.8 (9.0/13.3)
**5**		2.2/3.1 (2.3/3.3)	-/- (-/6.6)	5.4/8.7 (-/8.2)	6.1/10.0 (7.0/10.6)
**6**		3.8/5.4 (4.0/-)	-/8.3 (-/8.7)	5.6/8.7 (5.3/8.1)	4.8/8.0 (5.2/8.4)
**7**		3.2/4.3 (3.3/5.3)	-/- (-/8.8)	6.5/10.3 (9.0/10.2)	6.1/9.7 (6.5/10.7)
**11**		3.4/4.0 (-/-)	-/- (-/-)	8.6/12.3 (-/11.7)	10.7/15.5 (11.5/15.5)
**12**		6.2/7.8 (-/9.4)	-/- (-)	6.5/8.9 (-/9.1)	9.0/13.1 (10.4/14.7)
dihedral angle α (Scheme 4), in ^o^					
**5**	vacuum	22 (16)	- (-)	180 (-)	180 (160)
	toluene	24 (15)	- (-)	180 (-)	173 (157)
	acetonitrile	25 (15)	- (42)	180 (163)	164 (155)
**6**	vacuum	19 (13)	- (-)	179 (169)	176 (160)
	toluene	21 (-)	- (39)	179 (167)	174 (157)
	acetonitrile	24 (-)	2 (20)	179 (166)	169 (154)
**7**	vacuum	23 (15)	- (-)	180 (-)	176 (163)
	toluene	24 (15)	- (-)	179 (-)	168 (161)
	acetonitrile	26 (15)	- (35)	178 (169)	162 (160)
**11**	vacuum	22 (-)	- (-)	180 (-)	173 (159)
	toluene	23 (-)	- (-)	180 (-)	171 (160)
	acetonitrile	24 (-)	- (-)	179 (157)	168 (165)
**12**	vacuum	22 (-)	- (-)	180 (-)	179 (160)
	toluene	23 (-)	- (-)	180 (-)	176 (159)
	acetonitrile	25 (17)	- (-)	180 (180)	169 (160)

## Supplementary Materials

The following are available online at https://www.mdpi.com/2072-666X/11/10/901/s1, Figure S1: Comparison between the excited (S_1_) state energy landscapes (in relative energies in kcal/mol units) of of **5** obtained by CAM-B3LYP/TZVP (a) and M06-2X/TZVP (b, the same as in Figure 2b) in vacuum (green), toluene (red), acetonitrile (blue) and formamide (violet). The filled circles represent optimized structures.; Figure S2: Predicted absorption spectra (B3LYP/TZVP//M06-2X/TZVP) in toluene (red) and in acetonitrile (blue) of the different tautomers of **4–7** (a–d) and of **11–12** (e–f): **E1K**—solid line, **K2E—**dashes, **K2K**—dots, **K1K** (only in **6** in acetonitrile) – black dots. The spectra in formamide are practically identical to those in acetonitrile.; Figure S3: Ground state energy landscape (change of the relative energies in kcal/mol units) of **8**–**10** (a–c) and **13** (d) in vacuum (green), toluene (red), acetonitrile (blue) and formamide (violet). The filled circles represent optimized structures.;, Table S1: Frank-Condon states (vertical transitions) of the **E1K** form of **4**–**7** and **11**–**12**.; Table S2: Important structural parameters of the tautomers of **4**–**7** and **11–12** in ground and excited S_1_ (in brackets) state.; Table S3: Natural charges in vacuum in selected atoms in **4** and **5** in ground state and excited state (in brackets).; Table S4: Milliken atomic charges in vacuum in selected atoms in ground state.; Scheme S1: Ground state NBO charges of the different tautomers of **4–7** and of **11–12**. The donor (in blue)–acceptor (in red) interactions in the molecular backbones are presented by summing the natural charges of the different parts in the molecules.

## References

[1] Hynes J.T., Klinman J.P., Limbach H.-H., Schowen R.L. (2006). Hydrogen-Transfer Reactions.

[2] Antonov L. (2014). Tautomerism: Methods and Theories.

[3] Stasyuk A.J., Cywiński P.J., Gryko D.T. (2016). Excited-state intramolecular proton transfer in 2′-(2′-hydroxyphenyl)imidazo[1,2- a ]pyridines. J. Photochem. Photobiol. C Photochem. Rev..

[4] Serdiuk I.E., Roshal A.D. (2017). Exploring double proton transfer: A review on photochemical features of compounds with two proton-transfer sites. Dyes Pigments.

[5] Demchenko A.P., Tang K.-C., Chou P.-T. (2013). Excited-state proton coupled charge transfer modulated by molecular structure and media polarization. Chem. Soc. Rev..

[6] Kwon J.E., Park S.Y. (2011). Advanced Organic Optoelectronic Materials: Harnessing Excited-State Intramolecular Proton Transfer (ESIPT) Process. Adv. Mater..

[7] Tsutsui M., Taniguchi M. (2012). Single Molecule Electronics and Devices. Sensors.

[8] Sakai K., Tsuzuki T., Itoh Y., Ichikawa M., Taniguchi Y. (2005). Using proton-transfer laser dyes for organic laser diodes. Appl. Phys. Lett..

[9] Chen K.-Y., Hsieh C.-C., Cheng Y.-M., Lai C.-H., Chou P.-T. (2006). Extensive spectral tuning of the proton transfer emission from 550 to 675 nm via a rational derivatization of 10-hydroxybenzo[h]quinoline. Chem. Commun..

[10] Smith T.P., Zaklika K.A., Thakur K., Walker G.C., Tominaga K., Barbara P.F. (1992). Ultrafast studies on proton transfer in photostabilizers. J. Photochem. Photobiol. Chem..

[11] Irie M., Fukaminato T., Matsuda K., Kobatake S. (2014). Photochromism of Diarylethene Molecules and Crystals: Memories, Switches, and Actuators. Chem. Rev..

[12] Nedeltcheva-Antonova D., Antonov L., Antonov L. (2016). Controlled Tautomerism: Is It Possible?. Tautomerism: Concepts and Applications in Science and Technology.

[13] Kassem S., van Leeuwen T., Lubbe A.S., Wilson M.R., Feringa B.L., Leigh D.A. (2017). Artificial molecular motors. Chem. Soc. Rev..

[14] Kassem S., Lee A.T.L., Leigh D.A., Markevicius A., Solà J. (2015). Pick-up, transport and release of a molecular cargo using a small-molecule robotic arm. Nat. Chem..

[15] Jalink C.J., van Ingen W.M., Huizer A.H., Varma C.A.G.O. (1991). Prospects for using photoinduced intramolecular proton transfer to study the dynamics of conformational changes in flexible molecular chains. J. Chem. Soc. Faraday Trans..

[16] Jalink C.J., Huizer A.H., Varma C.A.G.O. (1992). Rate-limiting action of a proton crane in long-range intramolecular proton transfer. J. Chem. Soc. Faraday Trans..

[17] de Bekker E.J.A., Geerlings J.D., Varma C.A.G.O. (2000). Mechanism of a Photoinduced Solvent-Assisted Transfer of a Proton to a Specified Remote Target. J. Phys. Chem. A.

[18] de Bekker E.J.A., Pugzlys A., Varma C.A.G.O. (2001). Elementary Processes in Photoinduced Proton Transfers in 2-Hydroxy-1-( *N* -morpholinomethyl)naphthalene and 7-Hydroxy-8-( *N* -morpholinomethyl)quinoline in Liquid Solutions. J. Phys. Chem. A.

[19] van der Loop T.H., Ruesink F., Amirjalayer S., Sanders H.J., Buma W.J., Woutersen S. (2014). Unraveling the Mechanism of a Reversible Photoactivated Molecular Proton Crane. J. Phys. Chem. B.

[20] Wu K.-C., Cheng Y.-M., Lin Y.-S., Yeh Y.-S., Pu S.-C., Hu Y.-H., Yu J.-K., Che P.-T. (2004). Competitive intramolecular hydrogen bonding formation and excited-state proton transfer reaction in 1-[(diethylamino)-methyl]-2-hydroxy-3-naphthaldehyde. Chem. Phys. Lett..

[21] Wu K.-C., Lin Y.-S., Yeh Y.-S., Chen C.-Y., Ahmed M.O., Chou P.-T., Hon Y.-S. (2004). Design and synthesis of intramolecular hydrogen bonding systems. Their application in metal cation sensing based on excited-state proton transfer reaction. Tetrahedron.

[22] Antonov L., Deneva V., Simeonov S., Kurteva V., Nedeltcheva D., Wirz J. (2009). Exploiting Tautomerism for Switching and Signaling. Angew. Chem. Int. Ed..

[23] Antonov L.M., Kurteva V.B., Simeonov S.P., Deneva V.V., Crochet A., Fromm K.M. (2010). Tautocrowns: A concept for a sensing molecule with an active side-arm. Tetrahedron.

[24] Nedeltcheva D., Kurteva V., Antonov L. (2011). Gas-phase study of molecular switches based on tautomeric proton transfer. Eur. J. Mass Spectrom..

[25] Courtot P., Pichon R., Le Saint J. (1976). Determination du site de chelation chez les arylhydrazones de tricetones et D’ α-dicetones substituees. Tetrahedron Lett..

[26] Courtot P., Pichon R., Le Saint J. (1976). Photochromisme par isomerisation syn-anti de phenylhydrazones-2- de tricetones-1,2,3 et de dicetones-1,2 substituees. Tetrahedron Lett..

[27] Courtot P., Pichon R., Le Saint J. (1979). Échanges inter et intra-molćulaires du proton entre deux atomes d’azote de cycles chélatés hydrazone-imine et azo-énamine. Tetrahedron Lett..

[28] Pichon R., Le Saint J., Courtot P. (1981). Photoisomerisation d’arylhydrazones-2 de dicetones-1,2 substituees en 2. Tetrahedron.

[29] Yoder C.H., Barth R.C., Richter W.M., Snavely F.A. (1972). Nuclear magnetic resonance study of some nitrogen-15 substituted azo heterocycles. J. Org. Chem..

[30] Shawali A.S., Harb N.M.S., Badahdah K.O. (1985). A study of tautomerism in diazonium coupling products of 4-hydroxycoumarin. J. Heterocycl. Chem..

[31] Yordanov D., Deneva V., Georgiev A., Vassilev N., Vala M., Zhivkov I., Antonov L. (2020). 4-OH coumarin based rotary switches: Tautomeric state and effect of the stator. Dyes Pigments.

[32] Su X., Aprahamian I. (2014). Hydrazone-based switches, metallo-assemblies and sensors. Chem. Soc. Rev..

[33] Tatum L.A., Su X., Aprahamian I. (2014). Simple Hydrazone Building Blocks for Complicated Functional Materials. Acc. Chem. Res..

[34] Foy J.T., Ray D., Aprahamian I. (2015). Regulating signal enhancement with coordination-coupled deprotonation of a hydrazone switch. Chem. Sci..

[35] Harris J.D., Moran M.J., Aprahamian I. (2018). New molecular switch architectures. Proc. Natl. Acad. Sci. USA.

[36] Sobolewski A.L. (2008). Reversible molecular switch driven by excited-state hydrogen transfer. Phys. Chem. Chem. Phys..

[37] Lapinski L., Nowak M.J., Nowacki J., Rode M.F., Sobolewski A.L. (2009). A Bistable Molecular Switch Driven by Photoinduced Hydrogen-Atom Transfer. ChemPhysChem.

[38] Rode M.F., Sobolewski A.L. (2010). Effect of Chemical Substituents on the Energetical Landscape of a Molecular Photoswitch: An Ab Initio Study. J. Phys. Chem. A.

[39] Vetokhina V., Nowacki J., Pietrzak M., Rode M.F., Sobolewski A.L., Waluk J., Herbich J. (2013). 7-Hydroxyquinoline-8-carbaldehydes. 1. Ground- and Excited-State Long-Range Prototropic Tautomerization. J. Phys. Chem. A.

[40] Csehi A., Illés L., Halász G.J., Vibók Á. (2013). The effect of chemical substituents on the functionality of a molecular switch system: A theoretical study of several quinoline compounds. Phys. Chem. Chem. Phys..

[41] Csehi A., Woywod C., Halász G., Vibók Á. (2013). Ab initio studies of two pyrimidine derivatives as possible photo-switch systems. Open Phys..

[42] Csehi A., Halász G.J., Vibók Á. (2014). Molecular switch properties of 7-hydroxyquinoline compounds. Int. J. Quantum Chem..

[43] Ortiz-Sánchez J.M., Gelabert R., Moreno M., Lluch J.M., Anglada J.M., Bofill J.M. (2010). Bipyridyl Derivatives as Photomemory Devices: A Comparative Electronic-Structure Study. Chem.-Eur. J..

[44] Kaczmarek L.S. (1985). Bipyridines. Part XVIII. On the Synthesis of [2,2’-Bipyridine]-3-ol and Other Novel 2,2’-Bipyridine Derivatives from [2,2’-Bipyridine]-3,3’-diamine. Bull. Pol. Acad. Sci. Tech. Sci..

[45] Kaczmarek Ł, Balicki R., Lipkowski J., Borowicz P., Grabowska A. (1994). Structure and photophysics of deazabipyridyls. Excited internally hydrogen-bonded systems with one proton transfer reaction site. J. Chem. Soc. Perkin Trans. 2.

[46] Borowicz P., Grabowska A., Leś A., Kaczmarek Ł., Zagrodzki B. (1998). New phototautomerizing systems: Non-symmetric derivatives of [2,2′-bipyridyl]-3,3′-diol. Chem. Phys. Lett..

[47] Kaczmarek Ł., Zagrodzki B., Kamieński B., Pietrzak M., Schilf W., Leś A. (2000). Synthesis and NMR study of new derivatives of [2,2′-bipyridyl]-3,3′-diol and [2,2′-bipyridyl]-3-ol. J. Mol. Struct..

[48] Böhnke H., Bahrenburg J., Ma X., Röttger K., Näther C., Rode M.F., Sobolewski A.L., Temps F. (2018). Ultrafast dynamics of the ESIPT photoswitch *N*-(3-pyridinyl)-2-pyridinecarboxamide. Phys. Chem. Chem. Phys..

[49] Antonov L. (2020). Favipiravir tautomerism: A theoretical insight. Theor. Chem. Acc..

[50] Antonov L. (2019). Tautomerism in Azo and Azomethyne Dyes: When and If Theory Meets Experiment. Molecules.

[51] Deneva V., Vassilev N.G., Hristova S., Yordanov D., Hayashi Y., Kawauchi S., Fennel F., Völzer T., Lochbrunner S., Antonov L. (2020). Chercher de l’eau: The switching mechanism of the rotary switch ethyl-2-(2-(quinolin-8-yl)hydrazono)-2-(pyridin-2-yl)acetate. Comput. Mater. Sci..

[52] Manolova Y., Marciniak H., Tschierlei S., Fennel F., Kamounah F.S., Lochbrunner S., Antonov L. (2017). Solvent control of intramolecular proton transfer: Is 4-hydroxy-3-(piperidin-1-ylmethyl)-1-naphthaldehyde a proton crane?. Phys. Chem. Chem. Phys..

[53] Marciniak H., Hristova S., Deneva V., Kamounah F.S., Hansen P.E., Lochbrunner S., Antonov L. (2017). Dynamics of excited state proton transfer in nitro substituted 10-hydroxybenzo[h]quinolines. Phys. Chem. Chem. Phys..

[54] Frisch M.J., Trucks G.W., Schlegel H.B., Scuseria G.E., Robb M.A., Cheeseman J.R., Scalmani G., Barone V., Mennucci B., Petersson G.A. (2013). Gaussian 09 Revision D.01.

[55] Zhao Y., Truhlar D.G. (2008). Density Functionals with Broad Applicability in Chemistry. Acc. Chem. Res..

[56] Zhao Y., Truhlar D.G. (2008). The M06 suite of density functionals for main group thermochemistry, thermochemical kinetics, noncovalent interactions, excited states, and transition elements: Two new functionals and systematic testing of four M06-class functionals and 12 other functionals. Theor. Chem. Acc..

[57] Schäfer A., Huber C., Ahlrichs R. (1994). Fully optimized contracted Gaussian basis sets of triple zeta valence quality for atoms Li to Kr. J. Chem. Phys..

[58] Kawauchi S., Antonov L. (2013). Description of the Tautomerism in Some Azonaphthols. J. Phys. Org. Chem..

[59] Antonov L., Kurteva V., Crochet A., Mirolo L., Fromm K.M., Angelova S. (2012). Tautomerism in 1-phenylazo-4-naphthols: Experimental results vs quantum-chemical predictions. Dyes Pigments.

[60] Angelova S., Paskaleva V., Kochev N., Antonov L. (2019). DFT study of hydrazone-based molecular switches: The effect of different stators on the on/off state distribution. Mol. Phys..

[61] Fang H., Kim Y. (2017). Hydrogen-bonded channel-dependent mechanism of long-range proton transfer in the excited-state tautomerization of 7-hydroxyquinoline: A theoretical study. Theor. Chem. Acc..

[62] Mori Y. (2018). Reaction pathway and H/D kinetic isotope effects of the triple proton transfer in a 7-hydroxyquinoline-methanol complex in the ground state: A computational approach. J. Phys. Org. Chem..

[63] Tomasi J., Mennucci B., Cammi R. (2005). Quantum Mechanical Continuum Solvation Models. Chem. Rev..

[64] Schlegel H.B. (1982). Optimization of equilibrium geometries and transition structures. J. Comput. Chem..

[65] Improta R., Barone V. (2011). UV-Visible Absorption and Emission Energies in Condensed Phase by PCM/TD-DFT Methods. Computational Strategies for Spectroscopy.

[66] Adamo C., Jacquemin D. (2013). The calculations of excited-state properties with Time-Dependent Density Functional Theory. Chem. Soc. Rev..

[67] Antonov L., Kawauchi S., Okuno Y. (2014). Prediction of the color of dyes by using time-dependent density functional theory. Bulg. Chem. Commun..

[68] Yanai T., Tew D.P., Handy N.C. (2004). A new hybrid exchange–correlation functional using the Coulomb-attenuating method (CAM-B3LYP). Chem. Phys. Lett..

[69] Becke A.D. (1993). Density-functional thermochemistry. III. The role of exact exchange. J. Chem. Phys..

[70] Weinhold F., Landis C.R. (2005). Valency and Bonding: A Natural Bond Orbital Donor-Acceptor Perspective.

[71] Konijnenberg J., Ekelmans G.B., Huizer A.H., Varma C.A.G.O. (1989). Mechanism and solvent dependence of the solvent-catalysed pseudo-intramolecular proton transfer of 7-hydroxyquinoline in the first electronically excited singlet state and in the ground state of its tautomer. J. Chem. Soc. Faraday Trans 2.

[72] Al-Lawatia N., Husband J., Steinbrecher T., Abou-Zied O.K. (2011). Tautomerism in 7-Hydroxyquinoline: A Combined Experimental and Theoretical Study in Water. J. Phys. Chem. A.

[73] Miura M., Harada J., Ogawa K. (2007). Temperature-Induced Reversal of Proton Tautomerism: Role of Hydrogen Bonding and Aggregation in 7-Hydroxyquinolines. J. Phys. Chem. A.

[74] Lee S.-I., Jang D.-J. (1995). Proton Transfers of Aqueous 7-Hydroxyquinoline in the First Excited Singlet, Lowest Triplet, and Ground States. J. Phys. Chem..

[75] Lavin A., Collins S. (1993). The ground-state stabilization of the keto tautomer of 7-hydroxyquinoline in methanol/argon matrices at 10 K. Chem. Phys. Lett..

[76] Itoh M., Adachi T. (1984). Transient absorption and two-step laser excitation fluorescence studies of the excited-state proton transfer and relaxation in the methanol solution of 7-hydroxyflavone. J. Am. Chem. Soc..

[77] Kang W.-K., Cho S.-J., Lee M., Kim D.-H., Ryoo R., Jung K.-H., Jang D.-J. (1992). Excited State Intramolecular Proton Transfer and Physical Properties of 7-Hydroxyquinoline. Bull. Korean Chem. Soc..

[78] Park S.-Y., Jang D.-J. (2010). Accumulated Proton-Donating Ability of Solvent Molecules in Proton Transfer. J. Am. Chem. Soc..

[79] Mason S.F., Philp J., Smith B.E. (1968). Prototropic equilibria of electronically excited molecules. Part II. 3-, 6-, and 7-Hydroxyquinoline. J. Chem. Soc. Inorg. Phys. Theor..

[80] Bardez E. (1999). Excited-State Proton Transfer in Bifunctional Compounds. Isr. J. Chem..

[81] Kwon O.-H., Lee Y.-S., Yoo B.K., Jang D.-J. (2006). Excited-State Triple Proton Transfer of 7-Hydroxyquinoline along a Hydrogen-Bonded Alcohol Chain: Vibrationally Assisted Proton Tunneling. Angew. Chem. Int. Ed..

[82] Park S.-Y., Kim B., Lee Y.-S., Kwon O.-H., Jang D.-J. (2009). Triple proton transfer of excited 7-hydroxyquinoline along a hydrogen-bonded water chain in ethers: Secondary solvent effect on the reaction rate. Photochem. Photobiol. Sci..

[83] Hoffmann F., Ekimova M., Bekçioğlu-Neff G., Nibbering E.T.J., Sebastiani D. (2016). Combined Experimental and Theoretical Study of the Transient IR Spectroscopy of 7-Hydroxyquinoline in the First Electronically Excited Singlet State. J. Phys. Chem. A.

[84] Ekimova M., Hoffmann F., Bekçioğlu-Neff G., Rafferty A., Kornilov O., Nibbering E.T.J., Sebastiani D. (2019). Ultrafast Proton Transport between a Hydroxy Acid and a Nitrogen Base along Solvent Bridges Governed by the Hydroxide/Methoxide Transfer Mechanism. J. Am. Chem. Soc..

[85] Kerdpol K., Daengngern R., Meeprasert J., Namuangruk S., Kungwan N. (2016). Theoretical insights into photoinduced proton transfer of 7-hydroxyquinoline via intermolecular hydrogen-bonded wire of mixed methanol and water. Theor. Chem. Acc..

[86] Partovi–Azar P., Sebastiani D. (2020). Optimized effective potentials to increase the accuracy of approximate proton transfer energy calculations in the excited state. J. Chem. Phys..

[87] Kamlet M.J., Abboud J.L.M., Taft R.W., Taft R.W. (1981). An Examination of Linear Solvation Energy Relationships. Progress in Physical Organic Chemistry.

[88] Kamlet M.J., Abboud J.L.M., Abraham M.H., Taft R.W. (1983). Linear solvation energy relationships. 23. A comprehensive collection of the solvatochromic parameters,.pi.*,.alpha., and.beta., and some methods for simplifying the generalized solvatochromic equation. J. Org. Chem..

[89] Joule J.A., Mills K. (2009). Heterocyclic Chemistry.

