# Supplementary material for: 8-(Pyridin-2-yl)quinolin-7-ol as a Platform for Conjugated Proton Cranes: A DFT Structural Design
Source: Micromachines (Basel). 2020 Sep 29;11(10):901. doi: 10.3390/mi11100901 (PMC7601234; doi:10.3390/mi11100901)
Supplement: Supplementary file 1 [file micromachines-11-00901-s001.pdf]

8-(pyridin-2-yl)quinolin-7-ol as a platform for conjugated proton cranes: a DFT structural design

Anton Georgiev and Liudmil Antonov

Table S1. Frank-Condon states (vertical transitions) of the **E1K** form of **4**-**7** and **11**-**12**.

**Compound**	Environment	Singlet excited state					
		S_1			S_2		
		E*, kcal/mol	*f*	origin	E*, kcal/mol	*f*	origin
**4**	vacuum	26	0.08	mainly HOMO-LUMO	27	0.0	mainly (HOMO-2)-LUMO
	toluene	27	0.13		30	0.0	
	acetonitrile	29	0.12		34	0.0	
**5**	vacuum	25	0.23	HOMO-LUMO	36	0.0	mainly (HOMO-2)-LUMO
	toluene	20	0.33		32	0.0	
	acetonitrile	21	0.30		32	0.0	
**6**	vacuum	20	0.21	HOMO-LUMO	30	0.0	mainly (HOMO-3)-LUMO
	toluene	19	0.29		30	0.0	
	acetonitrile	21	0.26		31	0.0	
**7**	vacuum	22	0.28	mainly HOMO-LUMO	32	0.08	mainly HOMO-(LUMO+1)
	toluene	20	0.40		31	0.12	
	acetonitrile	22	0.39		33	0.10	
**11**	vacuum	33	0.17	HOMO-LUMO	38	0.0	mixed
	toluene	28	0.21	mainly	36	0.0	
	acetonitrile	26	0.17	HOMO-LUMO	35	0.0	
**12**	vacuum	24	0.44	mainly HOMO-LUMO	30	0.0	mixed
	toluene	18	0.57		28	0.0	
	acetonitrile	16	0.54		26	0.0	

* relative energy in respect of the most stable optimized structure in S_1 (Table 1 and Figures 1-2).

Table S2. Important structural parameters of the tautomers of **4**-**7** and **11**-**12** in ground and excited S_1 (in brackets) state.

**Compound**	**Environment**	**E1K**	**K1K**	**K2K**	**K2E**
r_{XH} (X=O, $N_{1'}$ or N_{10}), in Å					
**4**	vacuum	0.963 (0.965)	-	-	1.009 (1.010)
	toluene	0.964 (0.967)	-	-	1.011 (1.010)
	acetonitrile	0.965 (0.968)	-	-	1.012 (1.011)
**5**	vacuum	0.996 (1.047)	- (-)	1.057 (-)	1.055 (1.024)
	toluene	0.998 (1.059)	- (-)	1.056 (-)	1.052 (1.024)
	acetonitrile	0.999 (1.076)	- (1.013)	1.056 (1.029)	1.043 (1.024)
**6**	vacuum	1.007	-	1.052	1.064

		(1.068)	(-)	(1.054)	(1.027)
	toluene	1.009<br>(-)	-<br>(1.018)	1.049<br>(1.039)	1.062<br>(1.027)
	acetonitrile	1.009<br>(-)	1.080<br>(1.023)	1.046<br>(1.031)	1.056<br>(1.027)
**7**	vacuum	0.993<br>(1.013)	-<br>()	1.060<br>()	1.049<br>(1.020)
	toluene	0.995<br>(1.015)	-<br>()	1.059<br>()	1.045<br>(1.021)
	acetonitrile	0.996<br>(1.017)	-<br>(1.017)	1.058<br>(1.033)	1.040<br>(1.023)
**11**	vacuum	0.991<br>(-)	-<br>(-)	1.061<br>(-)	1.044<br>(1.022)
	toluene	0.992<br>(-)	-<br>(-)	1.062<br>(-)	1.041<br>(1.026)
	acetonitrile	0.993<br>(-)	-<br>(-)	1.064<br>(1.030)	1.038<br>(1.080)
**12**	vacuum	0.993<br>(-)	-<br>(-)	1.065<br>(-)	1.049<br>(1.022)
	toluene	0.993<br>(-)	-<br>(-)	1.067<br>(-)	1.045<br>(1.026)
	acetonitrile	0.993<br>1.060	-<br>(-)	1.069<br>1.061	1.039<br>(1.046)

Table S3. Natural charges in vacuum in selected atoms in 4 and 5 in ground state* and excited state (in brackets).

**Comp.**	**E1K**			**K2K**			**K2E**		
	**O**	**N_{quin}**	**N_{pyr}**	**O**	**N_{quin}**	**N_{pyr}**	**O**	**N_{quin}**	**N_{pyr}**
**4**	−0.647<br>(−0.584)	−0.406<br>(−0.437)	-	-	-	-	−0.584<br>(−0.532)	−0.488<br>(−0.454)	-
**5**	−0.663<br>(−0.597)	−0.439<br>(−0.431)	−0.489<br>(−0.499)	−0.632<br>(−0.48)**	−0.519<br>(−0.36)**	−0.486<br>(−0.53)**	−0.619<br>(−0.569)	−0.492<br>(−0.484)	−0.513<br>(−0.470)
**6**	−0.665<br>(−0.595)	−0.443<br>(−0.450)	−0.525<br>(−0.524)	−0.648<br>(−0.574)	−0.520<br>(−0.486)	−0.509<br>(−0.512)	−0.628<br>(−0.576)	−0.492<br>(−0.488)	−0.546<br>(−0.508)
**7**	−0.667<br>(−0.621)	−0.438<br>(−0.480)	−0.463<br>(−0.470)	−0.645<br>(-)	−0.523<br>(-)	−0.464<br>(-)	−0.626<br>(−0.596)	−0.494<br>(−0.493)	−0.487<br>(−0.444)
**11**	−0.656<br>(-)	−0.443<br>(-)	−0.470<br>(-)	−0.610<br>(-)	−0.515<br>(-)	−0.486<br>(-)	−0.605<br>(−0.557)	−0.489<br>(−0.476)	−0.497<br>(−0.452)
**12**	−0.653<br>(-)	−0.443<br>(-)	−0.483<br>(-)	−0.608<br>(-)	−0.512<br>(-)	−0.485<br>(-)	−0.607<br>(−0.558)	−0.485<br>(−0.474)	−0.509<br>(−0.462)

* The corresponding value for the nitrogen atom in pyridine is −0.41; ** data for **TS2**, because **K2K** spontaneously relaxes to it.

Table S4. Milliken atomic charges in vacuum in selected atoms in ground state*.

**Comp.**	**E1K**			**K2K**			**K2E**		
	**O**	**N_{quin}**	**N_{pyr}**	**O**	**N_{quin}**	**N_{pyr}**	**O**	**N_{quin}**	**N_{pyr}**
**4**	−0.33	−0.08	-	-	-	-	−0.38	−0.28	-
**5**	−0.33	−0.16	−0.30	−0.44	−0.33	−0.22	−0.43	−0.21	−0.34
**6**	−0.33	−0.17	−0.32	−0.46	−0.33	−0.23	−0.43	−0.21	−0.35
**7**	−0.34	−0.16	−0.29	−0.45	−0.33	−0.17	−0.43	−0.22	−0.33
**11**	−0.32	−0.17	−0.28	−0.42	−0.33	−0.22	−0.41	−0.21	−0.32

**12**	−0.32	−0.17	−0.29	−0.42	−0.32	−0.23	−0.42	−0.20	−0.34

* The corresponding value for the nitrogen atom in pyridine is −0.13.

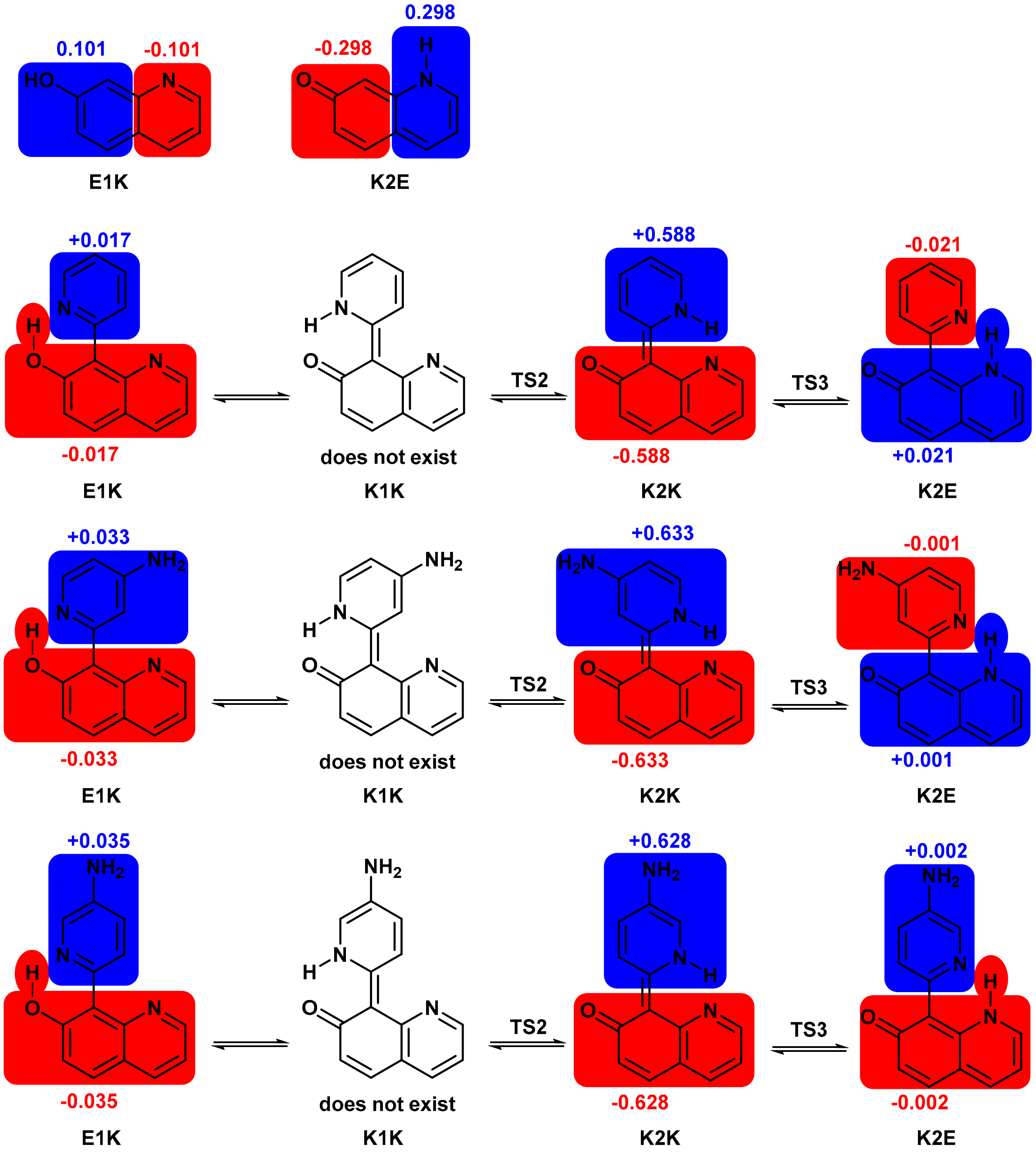

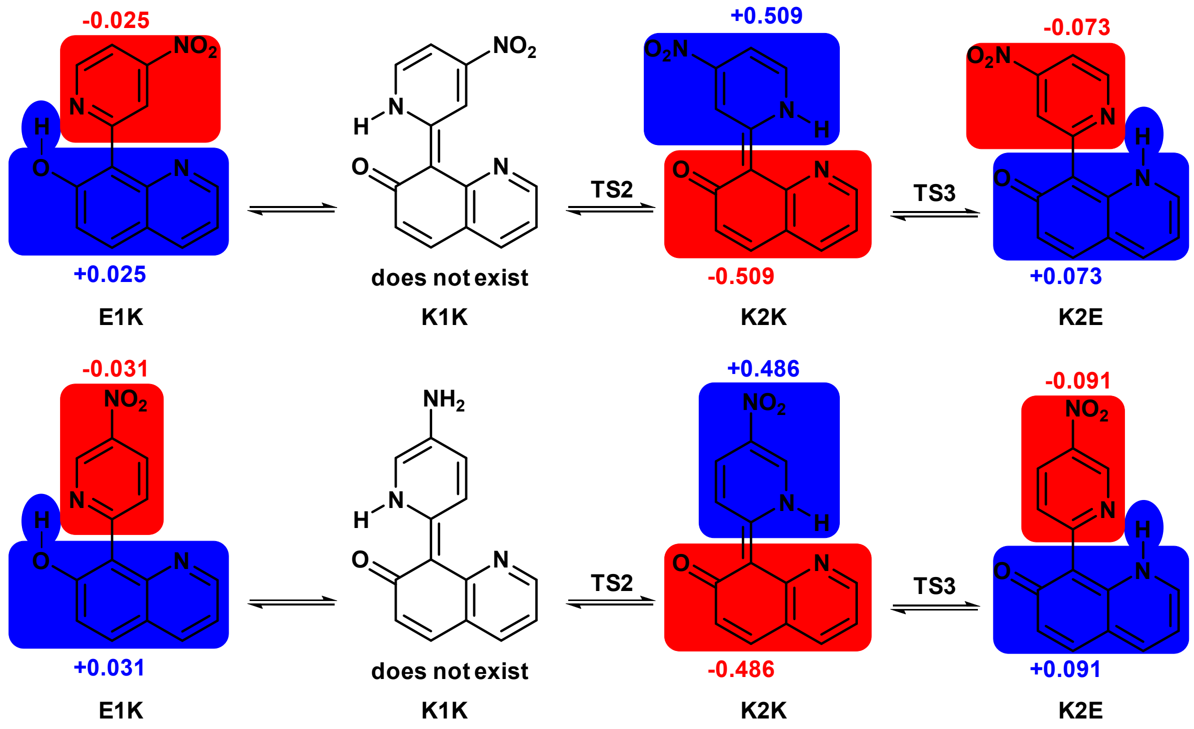

Scheme S1. Ground state NBO charges of the different tautomers of 4-7 and of 11-12. The donor (in blue) – acceptor (in red) interactions in the molecular backbones are presented by summing the natural charges of the different parts in the molecules.

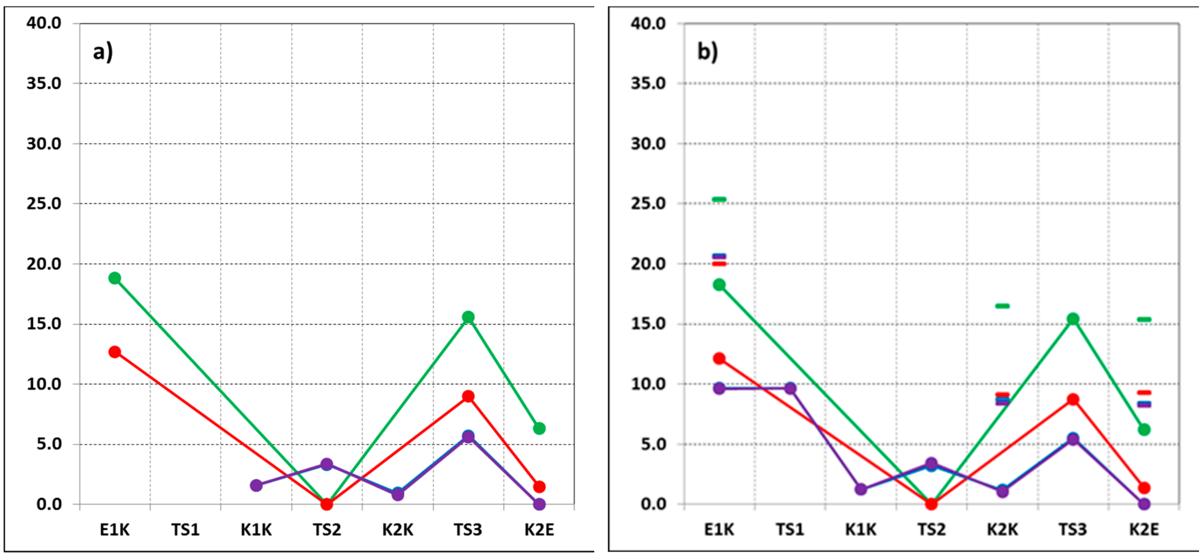

Figure S1. Comparison between the excited (S_1) state energy landscapes (in relative energies in kcal/mol units) of of **5** obtained by CAM-B3LYP/TZVP (**a**) and M06-2X/TZVP (**b**, the same as in Figure 2b) in vacuum (green), toluene (red), acetonitrile (blue) and formamide (violet). The filled circles represent optimized structures.

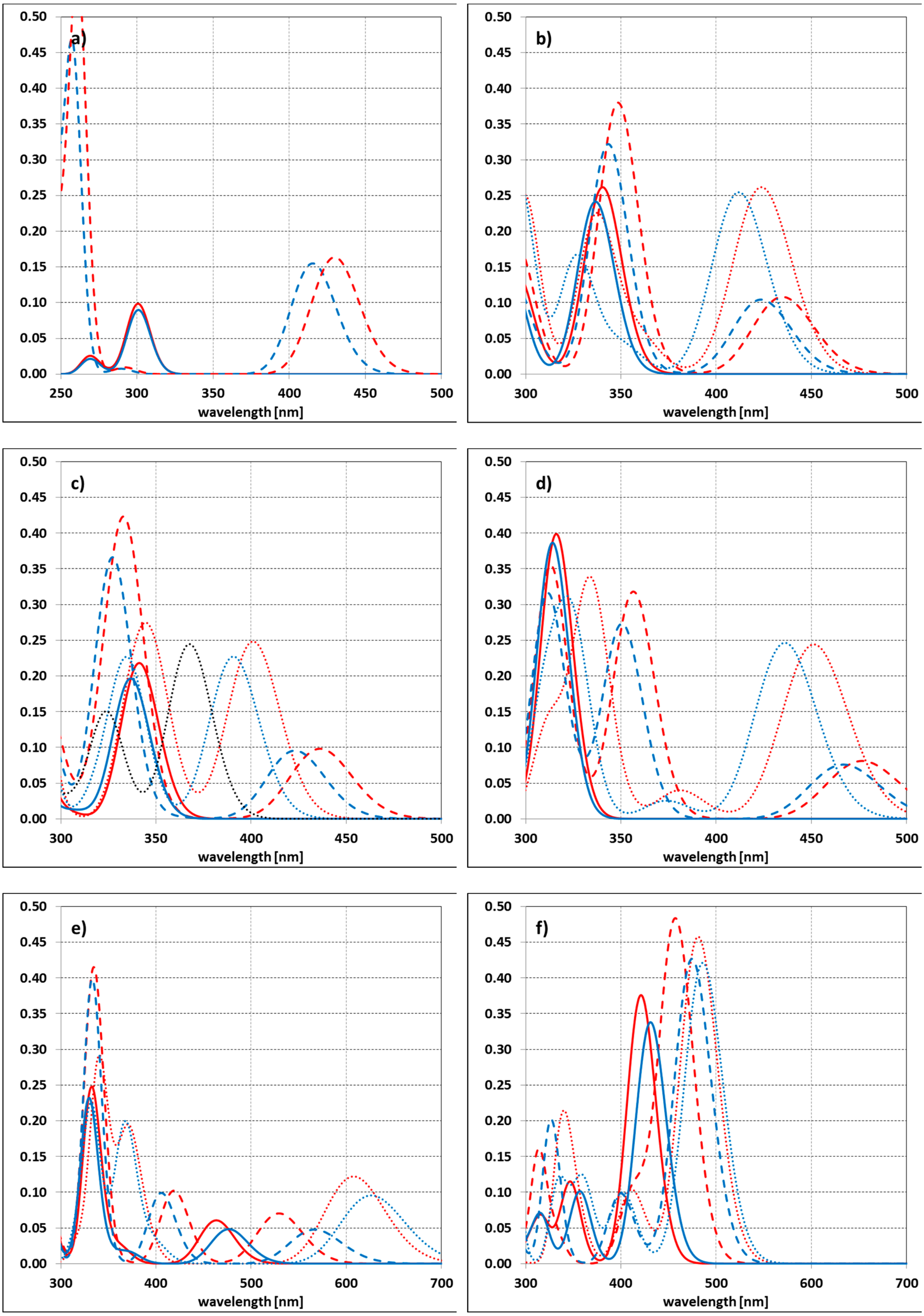

Figure S2. Predicted absorption spectra (B3LYP/TZVP//M06-2X/TZVP) in toluene (red) and in acetonitrile (blue) of the different tautomers of **4-7** (**a**–**d**) and of **11-12** (**e**–**f**): **E1K** – solid line, **K2E** – dashes, **K2K** – dots, **K1K** (only in **6** in acetonitrile) – black dots. The spectra in formamide are practically identical to those in acetonitrile.

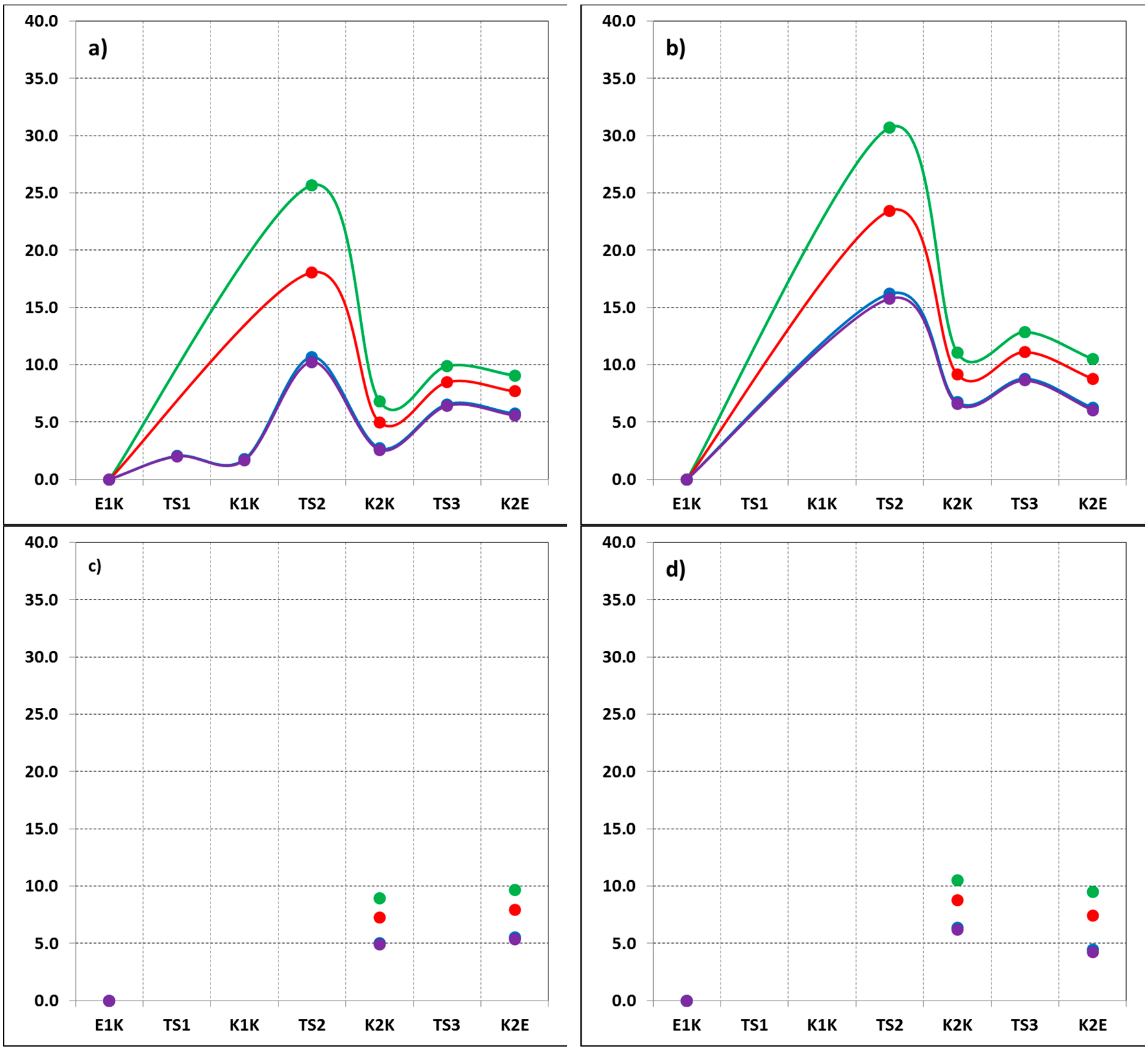

Figure S3. Ground state energy landscape (change of the relative energies in kcal/mol units) of **8-10** (**a**–**c**) and **13** (**d**) in vacuum (green), toluene (red), acetonitrile (blue) and formamide (violet). The filled circles represent optimized structures.
